# Genomic Insights into Unspecified Monogenic Forms of Diabetes and Their Associated Comorbidities: Implication for Treatment

**DOI:** 10.3390/cimb47121055

**Published:** 2025-12-17

**Authors:** Nadia Kheriji, Hamza Dallali, Mariem Gharbi, Asma Krir, Afef Bahlous, Melika Ben Ahmed, Faten Mahjoub, Abdelmajid Abid, Henda Jamoussi, Rym Kefi

**Affiliations:** 1 Laboratory of Biomedical Genomics and Oncogenetics, Institut Pasteur de Tunis, Tunis 1068, Tunisia; nadia.kheriji@pasteur.utm.tn (N.K.); hamza.dallali@pasteur.utm.tn (H.D.); m.gharbi.t@gmail.com (M.G.); abdelmajid.abid99@gmail.com (A.A.); 2 University of Tunis El Manar, Tunis 1006, Tunisia; faten_mahjoub@yahoo.fr (F.M.); hendajamoussi@gmail.com (H.J.); 3 Laboratory of Clinical Biochemistry and Hormonology, Institut Pasteur de Tunis, Tunis 1068, Tunisia; asma.krir@fmt.utm.tn (A.K.); afef_bahlous@yahoo.fr (A.B.); 4 Laboratory of Transmission, Control and Immunobiology of Infections (LR16IPT02), Department of Clinical Immunology, Institut Pasteur de Tunis, Tunis 1068, Tunisia; melika.benahmed@pasteur.tn; 5 Research Unit UR18ES01 on “Obesity”, Tunis 1007, Tunisia; 6 Department A, National Institute of Nutrition and Food Technology, Tunis 1007, Tunisia

**Keywords:** atypical diabetes, genetic variants, North Africa, bioinformatic analysis, healthcare management, genetic diagnosis

## Abstract

This study focuses on the genetic and clinical characterization of Monogenic Forms of Diabetes (MFD), which are frequently underdiagnosed or misclassified due to clinical similarities with type 1 and type 2 diabetes. Researchers performed Exome Sequencing on 11 Tunisian patients suspected of having MFD. The pathogenicity of genetic variants was assessed using filtering and bioinformatics prediction tools. The ORVAL online tool was used to predict the likelihood of combinations of pathogenic variants. Sanger sequencing confirmed likely pathogenic predicted variants in patients and assessed familial segregation. We identified 15 potentially pathogenic variants in 14 genes linked to MFD, including MODY-3, and isolated diabetes with low penetrance for Wolfram syndrome. Additionally, syndromic forms such as partial familial lipodystrophy types 2 and 4, and Wolfram syndrome were detected. Five patients exhibited characteristics of unspecified MFD. This study underscores the importance of genetic screening in individuals with diabetes who have a family history of the disease, particularly those with associated comorbidities. Our findings emphasize the crucial role of genetic testing in refining diabetes classification, leading to more accurate diagnoses. Therefore, integrating genetic research into clinical practice is essential to improving healthcare outcomes for patients with diabetes.

## 1. Introduction

Diabetes represents a major public health problem and socio-economic burden worldwide. It is characterized by chronic hyperglycaemia due to a defect in insulin secretion or insulin action, or a combination of both. Chronic hyperglycemia leads to degenerative organ complications over time, potentially leading to disabilities and death. Both the World Health Organization (WHO) and the International Diabetes Federation (IDF) consider diabetes a pandemic, as it currently affects over 580 million people globally and causes more than 3.4 million deaths per year [1].

According to the American Diabetes Association (ADA), diabetes is classified into four categories: (1) Type 1 diabetes (T1D, also known as insulin-dependent diabetes or juvenile diabetes) accounts for 5–10% of all diabetes cases. It involves the progressive destruction of pancreatic β-cells, occurring in a genetically susceptible background and associated with autoimmune manifestations. (2) Type 2 diabetes (T2D, also known as non-insulin-dependent or mature-onset diabetes) is the most common hyperglycemic state (90–95% of diabetics), primarily occurring in adults. It results from the combination of genetic and environmental factors. (3) Gestational diabetes is a form of hyperglycemia first recognized during pregnancy that may resolve postpartum but increases the long-term risk of T2D for both mother and child. (4) Other specific types of diabetes which include a heterogeneous group of conditions such as exocrine pancreatic diseases, endocrinopathies, drug-induced diabetes, and genetic forms.

Among the genetic causes, monogenic forms of diabetes (MFD) refer to diabetes resulting from single-gene defects that affect insulin production or secretion. It accounts for up to 5% of all diabetes cases and includes various subtypes such as neonatal diabetes, MODY (Maturity-Onset Diabetes of the Young), and syndromic forms. Due to its phenotypic overlap with T1D or T2D, MFD are often misclassified, leading to suboptimal treatment. Recognizing and accurately diagnosing monogenic diabetes is crucial, as it enables tailored therapeutic approaches and informed genetic counseling [2].

There are currently more than 50 subtypes of MFD including neonatal diabetes, the 14 subclasses of MODY diabetes (Maturity Onset Diabetes of the Young), syndromic forms of diabetes and other yet unknown subtypes [3].

Despite their clinical importance, MFD remain significantly underdiagnosed worldwide, especially in low- and middle-income countries. Several factors contribute to this under recognition such as:

(1) The phenotypic overlap between MFD, T1D, and T2D;
(2) The limited access to genetic testing and trained personnel;
(3) The lack of awareness among healthcare providers;
(4) The lack of standardized, easy-to-use screening tools in routine practice.

These challenges lead to diagnostic delays or misclassification, preventing patients from receiving targeted and effective treatment. Encouragingly, recent initiatives have aimed to fill this gap. For example, Welsch et al. developed a new clinical tool to help identify pediatric patients with atypical diabetes likely to be associated with genetic variants, offering a promising strategy to improve early detection and guide testing decisions in clinical settings [4].

However, MFD remains largely unexplored and poorly characterized in Tunisia where approximately 50% of individuals with diabetes are still undiagnosed [5].

The aim of the present study is threefold: first, to identify the genetic causes of MFD in Tunisian patients; second, to improve molecular diagnosis of MFD among the Tunisian population using next-generation sequencing (NGS) technologies, specifically, Whole Exome Sequencing (WES) and third, to inform treatment strategies that contributeto precision diagnosis and the advancement of personalized healthcare outcomes.

## 2. Materials and Methods

### 2.1. Patients and Sample Collection

A total of 24 Tunisian patients suspected of having atypical forms of diabetes were recruited from the Department of Nutritional Diseases at the National Institute of Nutrition and Food Technology (INNTA) based on the following inclusion criteria:

- Inclusion criteria

Fasting plasma glucose ≥ 1.26 g/L (7 mmol/L) [2].

Young age of diabetes onset (≤40 years).

A family history of diabetes spanning at least two generations.

To participate in this study, patients and available family members, including parents provided written informed consent. The study protocol was conducted in accordance with the *Declaration of Helsinki* and was ap-proved by the Ethical Committee of the Institut Pasteur of Tunis “IPT” (Registration numbers IRB00005445 and FWA00010074; ref. 2020/10/I/LR16IPT/V2).

Details regarding the medical histories of the patients and their extended family members (when available) were documented using a structured questionnaire. Additionally, clinical and metabolic data were collected from all patients, including the demographic information, anthropometric measurements, diabetes history, and treatment (oral anti-diabetic medications (OAD) and/or insulin injections). Biochemical parameters, such as fasting plasma glucose (FPG), glycated hemoglobin (HbA1c), C-peptide, lipid profile [total cholesterol (TC), triglycerides (TG), high-density lipoprotein (HDL), and low-density lipoprotein (LDL)], C-reactive protein (CRP), and creatinine were measured in collaboration with the Laboratory of Clinical Biochemistry and Hormonology in IPT.

Research on diabetes autoantibodies was carried out in collaboration with the Laboratory of Clinical Immunology in IPT. In our study, we analyzed titers of glutamic acid decarboxylase (GAD), islet antigen 2 (IA2), and islet cell antibodies (ICAs) in order to select suspected MD patients for genetic testing. In this study, we included the examination of the autoantibody titers among MD subjects selected for genetic testing as reported by Kheriji et al. [6].

Following comprehensive immunological and biochemical assessments, patients meeting any of the following exclusion criteria were excluded from the planned genetic testing:

- Exclusion criteria

Complete insulin deficiency (very low or undetectable C-peptide);

Presence of three or more pancreatic autoantibodies at high titers;

A clinical profile strongly suggestive of autoimmune diabetes.

This approach ensured the selection of 11 patients most likely to benefit from genetic testing for monogenic diabetes.

### 2.2. Genomic Investigation

#### 2.2.1. DNA Extraction

Genomic DNA was extracted from whole blood using the FlexiGene DNA Kit (QIAGEN, Germantown, MD, USA). DNA quality was then assessed using a NanoDrop spectrophotometer (Thermo Fisher Scientific, Waltham, MA, USA) and a DeNovix (Life Science Technologies, Carlsbad, CA, USA).

#### 2.2.2. WES

Whole Exome Sequencing (WES) analysis was performed in collaboration with RAN Bio Links SARL (Carthagenomics, Macrogen, Seoul, Republic of Korea) using the Sure Select Human All Exon V6 Kit (Agilent Technologies, Santa Clara, CA, USA) and the Twist Human Core Exo-me Kit (Twist Biosciences, San Francisco, CA, USA). The captured libraries were sequenced on NovaSeq 6000 System (Illumina, San Diego, CA, USA) to generate 151 bp paired-end reads.

#### 2.2.3. Bioinformatic Analysis

The quality of the sequencing reads in FASTQ files was evaluated using FastQC (https://www.bioinformatics.babraham.ac.uk/projects/fastqc/, accessed on 11 October 2025), which was followed by adapter trimming using BBDuk (https://jgi.doe.gov/data-and-tools/bbtools/bb-tools-user-guide/bbduk-guide/, accessed on 11 October 2025). We aligned reads to the human reference genome hg38, and we subsequently called the genetic variants in a VCF file following the GATK best practices. Variant annotation was processed using ANNOVAR (version 2019-10-24) [7]. As in our previous study, we applied the same analytical approach to prioritize potential disease-causing variants in the current investigation [6]. In fact, all identified variants were classified according to the American College of Medical Genetics and Genomics (ACMG) guidelines. To support variant interpretation, we applied a rigorous multi-criteria strategy combining in silico prediction tools, population allele frequency thresholds, genotype–phenotype correlation, and segregation analysis. This integrative framework allowed us to prioritize variants with plausible pathogenic potential, in alignment with ACMG/AMP standards. Additionally, we predicted the likelihood combinations of pathogenic variations using the machine-learning tool ORVAL (Oligogenic Resource for Variant AnaLysis: https://orval.ibsquare.be/, accessed on 11 October 2025). This platform uses a variety of variants, genes, and gene pair biological features to make predictions and create networks of potential pathogenic variant combinations in gene pairs rather than isolated variations in individual genes. ORVAL enables interactive result exploration, aiding biological interpretation and supporting deeper investigation of complex genetic diseases [8].

#### 2.2.4. Sanger Sequencing

Sanger sequencing was performed to confirm the likely pathogenic variants identified in the patients and to assess familial segregation using the ABI PRISM BigDye Terminator v3.1 Cycle Sequencing Kit (Thermo Fisher Scientific, Waltham, MA, USA).

## 3. Results

### 3.1. Cohort Study Description

**Figure 1:**
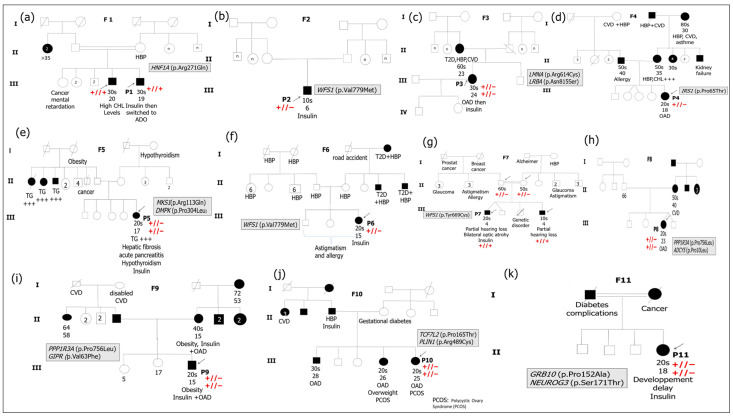
(**a**–**k**) Pedigrees of the 11 suspected MFD patients’ families. F indicates the proband’s family. P indicates the proband. The arrows indicate family members, whose DNA samples are available in the present study. Double horizontal lines indicate inbreeding. White squares and circles indicate healthy males and females, respectively. Black squares and circles indicate males and females with diabetes, respectively. The information below family members is ordered as follows: age at examination, age at diabetes diagnosis, clinical features, and/or specific anti-hyperglycemia treatment. OAD indicates oral antidiabetics. HBP refers to high blood pressure. CVD refers to Cardiovascular disease. TG refers to triglycerides. CHL +++ indicates hypercholesterolemia. Symbols + andor − indicate Sanger segregation information. +//+ indicates the presence of the variant in the homozygous state. +//− indicates the presence of the variant in the heterozygous state. −//− indicates the absence of the variant.

Based on biochemical and immunological results, only 11 Tunisian patients suspected of having monogenic forms of diabetes (MFD) were selected for genetic testing. The anthropometric and clinical characteristics of the participants are summarized in Table 1 while the results of biochemical measurements and immunological analyses are reported in Table 2. All patients demonstrated the absence of at least two measured pancreatic antibodies. The family history of diabetes along with additional information regarding clinical features are illustrated in Figure 1.

### 3.2. Genetic Findings

Our genetic investigation identified 15 potential pathogenic variants located in 14 genes associated with MFD in the 11 suspected MFD patients. A detailed summary of all genetic findings from our cohort study is presented in Table 3.

**Table 3:** List of the filtered genetic variants identified in the 11 suspected MFD patients.

Patient ID	Gene & Exon	Refseq	Genetic Variant	Genotype	Consequence	dbSNP	gnomAD Frequency	Pathogenicity Score	References	Final Pathogenicity Assessment
P1	*HNF1A* exon 4	NM_000545.8	c.812G>A	Hom	p.Arg271Gln	rs779184183	8.031 × 10^−6^	13	Clin var (ID:449403)	Pathogenic
P2	*WFS1* exon 8	NM_001145853.1	c.2335G>A	Het	p.Val779Met	rs141328044	0.0017	11	Uniprot/Clin Var (ID: 45453)	Pathogenic
P3	*LMNA* exon 10	NM_170708.4	c.1840C>T	Het	p.Arg614Cys	rs142000963	0.0012	13	The present study	Likely to be pathogenic
	*LRBA* exon 20	NM_001199282.2	c.2444A>G	Het	p.Asn815Ser	rs140666848	0.0022	9	ClinVar (ID: 218542)	Likely to be pathogenic
P4	*IRS1* exon 1	NM_005544.3	c.193C>A	Het	p.Pro65Thr	rs149830479	8.774 × 10^−5^	8	The present study	Likely to be pathogenic
P5	*MKS1* exon 4	NM_001165927.1	c.338G>A	Het	p.Arg113Gln	rs202112856	0.0004	8	ClinVar (ID: 235814) ClinVar (ID:2440881)	Likely to be pathogenic
	*DMPK* exon 7	NM_001288765.1	c.911C>T	Het	p.Pro304Leu	rs200491028	0.0002	8		Likely to be pathogenic
P6	*WFS1* exon 8	NM_001145853.1	c.2335G>A	Het	p.Val779Met	rs141328044	0.0017	10	Uniprot/Clin Var (ID: 45453)	Pathogenic
P7	*WFS1* exon 8	NM_001145853.1	c.2006A>G	Hom	p.Tyr669Cys	rs1402999203	3.987 × 10^−6^	14	Uniprot/ClinVar (ID: 2576526)	Pathogenic
P8	*PPP1R3A* exon 4	NM_002711.4	c.2267C>T	Het	p.Pro756Leu	rs151310594	0.0009	11	ClinVar (ID: 393402)	Benign
	*ADCY5* exon 1	NM_183357.3	c.29C>T	Het	p.Pro10Leu	rs143905423	0.0012	11	ClinVar (ID: 1170029)	Likely to be pathogenic
P9	*PPP1R3A* exon 4	NM_002711.4	c.2267C>T	Het	p.Pro756Leu	rs151310594	0.0009	11	ClinVar (ID: 393402)	Benign
	*GIPR* exon 4	NM_001308418.2	c.187G>T	Het	p.Val63Phe	rs142528936	5.171 × 10^−6^	14	The present study	Likely to be pathogenic
P10	*TCF7L2* exon 5	NM_001146283.2	c.493C>A	Het	p.Pro165Thr	-	-	12	The present study	Likely to be pathogenic
	*PLIN 1* exon 9	NM_001145311.2	c.1465C>T	Het	p.Arg489Cys	rs780710485	0.0002	8	The present study	Likely to be pathogenic
P11	*GRB 10* exon 4	NM_001001549.3	c.454C>G	Het	p.Pro152Ala	rs200175899	0.0008	10	The present study	Likely to be pathogenic
	*NEUROG3* exon2	NM_020999.4	c.511T>A	Het	p.Ser171Thr	rs200417293	0.0004	9	The present study	Likely to be pathogenic

The genetic results for patient P1 revealed a homozygous pathogenic variant c.812G>A;p.Arg271Gln in the *HNF1A* gene (ClinVar ID: 449403). This variant was also confirmed in the brother diagnosed with diabetes using sanger sequencing. WES analysis identified a heterozygous variant c.2335G>A;p.Val779Met in the *WFS1* gene in patients P2 and P6. This variant is considered pathogenic by Uniprot and as a variant of uncertain significance (VUS) in ClinVar (ID: 45453). Our genetic investigation of patient P3 uncovered two heterozygous variants: c.1840C>T;p.Arg614Cys in the *LMNA* gene and c.2444A>G;p.Asn815Ser in the *LRBA* gene. The LMNA variant is new and classified as VUS according to the ACMG criteria. Similarly, the LRBA variant is also classified as a VUS in ClinVar (ID: 218542). Querying the ORVAL tool confirmed the potential pathogenic effect of the *LMNA-LRBA* combination with 99% confidence, suggesting digenic interaction between the two variants. Patient P4 was found to be heterozygous for a new mutation c.193C>A;p.Pro65Thr in the *IRS1* gene. This variant is very rare (gnomAD f = 0.0000877) and classified as a VUS according to ACMG criteria. Genetic results for patient P5 showed the presence of two heterozygous variants: c.338G>A;p.Arg113Gln in the *MKS1* gene and c.911C>T;p.Pro304Leu in the *DMPK* gene. Both variants are categorized as VUSs in ClinVar (IDs: 235814 and 2440881), with pathogenicity scores of 8 out of 14 from various pathogenicity prediction tools. The ORVAL tool revealed no potential genetic combinations associated with these variants. A homozygous variant located in the *WFS1* gene was identified in P7 and confirmed in the brother, who was also diagnosed with diabetes, as well as in parents, who were heterozygous for the variant. This variant c.2006A>G;p.Tyr669Cys is classified as pathogenic according to ACMG criteria, ClinVar (ID: 2576526) and Uniprot. Patients P8 and P9 both carry a common variant c.2267C>T;p.Pro756Leu in the *PPP1R3A* gene, which is considered benign in ClinVar (ID: 393402) and ACMG criteria. Additionally, patient P8 has another variantc.29C>T, p.Pro10Leu in the *ADCY5* gene which is considered as likely pathogenic in ClinVar (ID: 1170029). The ORVAL analysis indicates a digenic effect of the *PPP1R3A-ADCY5* combination at 99% confidence. In contrast, P9 presents a new heterozygous variant c.187G>T;p.Val63Phe in the *GIPR* gene. Based on our genetic evaluation, this variant could potentially be pathogenic. The ORVAL tool shows a low pathogenicity score for the *PPP1R3A-GIPR* combination (VarCoPP score = 0.67). Two new variants c.493C>A;p. Pro165Thr and c.1465C>T;p.Arg489Cys were identified in P10, respectively, in the *TCF7L2* and *PLIN1* genes. According to our variant filtering and prioritization strategy, these variants are considered potentially pathogenic. The ORVAL tool indicates a dual molecular diagnosis involving the *TCF7L2-PLIN1* combination (VarCoPP score = 0.78, 99%). Our WES results led to the identification of two new variantsc.454C>G;p.Pro152Ala and c.511T>A;p.Ser171Thr in P11, located, respectively, in the *GRB10* and *NEUROG3* genes. Both variants are deemed potentially pathogenic. The ORVAL tool indicates that the combination of *GRB10-NEUROG3* genes variants results in a monogenic disease with a modifying effect (VarCoPP score = 0.80, 99%).

Summarize the results from the ORVAL tool, illustrating the potential pathogenic combinations among the variants identified in the studied patients. The results of Sanger sequencing for all index cases and any available family members are presented in Figure 1, Table 4.

### 3.3. Classification and Management of MFD

Table 5 summarizes all the MFD subclasses identified in the study cohort. These genetic results were communicated to the treating physicians to tailor treatment strategies for improved diabetes management. Among the 11 patients studied, genetic analysis allowed us to adjust and guide treatment decisions for four individuals.

## 4. Discussion

This study is among the first comprehensive WES-based investigations of MFD in the Tunisian population, integrating several pivotal elements: it provides population-specific insights, identifies both known and novel variants, and explores potential digenic or modifier effects using the ORVAL platform, refining our understanding of MFD’s complex genetic architecture. Notably, the findings had direct clinical translation, demonstrating the value of genetic diagnosis in guiding personalized care, especially in resource-limited settings. To evaluate the pathogenicity of the identified variants, we implemented a rigorous multi-step strategy combining complementary approaches: (i) In Silico prediction using 14 different tools to assess functional impact, (ii) segregation analysis within families to establish inheritance patterns, (iii) population frequency assessment using GnomAD and GME Variome databases, and (iv) genotype–phenotype correlation through integration of clinical and molecular data. This integrative approach reinforced the robustness of our findings and underscores the importance of close collaboration between clinicians and geneticists to refine variant interpretation and improve diagnostic accuracy.

### 4.1. MFD: MODY & Syndromic Diabetes

The MODY_3 (*HNF1A*) subtype was identified in the F1 family following the discovery of a homozygous mutation (c.812G>A) in the *HNF1A* gene in P1 and his diabetic brother. This mutation results in an amino acid substitution from arginine to glutamine at codon 271 (p. Arg271Gln). According to the literature, this mutation has been found in 19 unrelated individuals with non-autoimmune, insulin-deficient diabetes [9]. Functional studies indicate that the p.Arg271Gln protein alters DNA-binding capacity and reduces transactivation to 50–60% of the wild-type level [10]. Consequently, the p.Arg271Gln variant meets the criteria for classification as pathogenic according to the ACMG/AMP guidelines.

The absence of diabetes in both parents, supports findings from a previous study, which reported the presence of this variant in the heterozygous state in both healthy parents of the index case, indicating reduced penetrance of DM [11]. Regarding treatment, current guidelines recommend switching from insulin to sulfonylureas for patients with *HNF1A* mutation (*HNF1A*-MODY). Based to our genetic findings, the referring physicians advised patient P1 and the brother to stop insulin and instead use a combination of sulfonylureas (Glimid and Amarel) [12]. This therapeutic adjustment led to an improvement in glycated hemoglobin levels, which decreased from 9.01% to 7.7% in both Patient P1 and his brother (HbA1c from 7.12 to 6.3%).

The same variant identified in patients P2 and P6 has been previously reported in a Chinese woman with diabetes, diagnosed with Wolfram syndrome (WS) [13]. Dominant mutations in the *WFS1* gene have been shown to cause less severe phenotypes compared to recessive mutations associated with classical WS. In particular, heterozygous mutations in the *WFS1* gene have been linked to isolated adult-onset diabetes with low penetrance for WS features such as optic atrophy, hearing impairment, and isolated congenital nuclear cataract [14,15]. These results underscore the significant clinical heterogeneity associated with variants in the *WFS1* gene. Early identification of deleterious mutations is crucial for the personalized management of patients with isolated diabetes and low penetrance for WS features, as it can improve their clinical prognosis [16]. Based on our genetic analysis, we recommend that patients P2 and P6 receive comprehensive, multidisciplinary healthcare management.

Genetic investigation in P3 revealed two heterozygous variants in the *LMNA* and *LRBA* genes. The LMNA protein plays an important role in nuclear assembly, chromatin organization, nuclear membrane integrity, and telomere dynamics. It is essential for the normal development of peripheral nervous system and skeletal muscle [17]. Our findings support previous studies that have demonstrating the impact of the *LMNA* variants on glucose homeostasis, particularly in individuals of African descent [18]. An independent analysis of variants in the *LMNA* gene via the T2D Knowledge Portal revealed that the p.G602S variant is significantly as-sociated with T2DM (*p* = 0.02; odds ratio = 4.58) in African-Americans (allele frequency 0.297%). Notably, a study correlated a novel variant (p.K108E) in the *LMNA* gene with psychiatric and metabolic diseases in Latinos [19], similar to our patient P3, who experiences psychiatric issues. According to the literature, mutations in the *LMNA* gene may be linked to Familial partial lipodystrophy type 2. Most individuals with this condition exhibit high triglycerides levels (hypertriglyceridemia) and insulin resistance as seen in P3 [20].

The *LRBA* (LPS-responsive beige-like anchorprotein) gene encodes a protein involved in immune system regulation. Mutations in this gene have been associated with a rare autoimmune disease called LRBA deficiency. According to the literature, LRBA deficiency is characterized by a wide range of clinical manifestations, including immune dysregulation, hypogammaglobulinemia (low levels of specific antibodies), recurrent infections, and autoimmune conditions, as seen in P3. T1DM is one of the autoimmune diseases that may be associated with LRBA deficiency [21]. However, the diagnosis of T1D was excluded due to the absence of pancreatic autoantibodies in P3. The ORVAL results showed a true digenic potentially pathogenic combination (VarCoPP score = 0.82) between the variants identified in *LMNA* and *LRBA* genes, suggesting that both genes contribute to the onset of diabetes in P3. Collectively, these findings point to an atypical, unspecified form of diabetes known as partial familial lipodystrophy type 2 in P3.

Patient P4, adolescent with diabetes, carrying a heterozygous mutation (c.193C>A:p.P65T) in the *IRS1* gene. Insulin Receptor Substrate 1 (IRS1) encodes a protein that is phosphorylated by the insulin receptor tyrosine kinase. Mutations in this gene have been linked to T2D [22,23]. Therefore, the genetic investigation in P4 support a diagnosis of T2D at a young age.

Genetic investigation in P7 revealed a homozygous pathogenic variant (c.2006A>G;p.Tyr669Cys) in the *WFS1* gene. This variant is a missense mutation that results in an amino acid substitution from a large, aromatic residue (tyrosine) to a medium-sized, polar residue (Cysteine). As previously mentioned, mutations in the *WFS1* gene are associated with WS, also known as DIDMOAD syndrome (Diabetes Insipidus, Diabetes Mellitus, Optic Atrophy, and Deafness). Patient P7 exhibits clinical features consistent with this condition, including, early–onset diabetes at the age of 4, partial hearing loss (sensorineural deafness), and bilateral optic atrophy. The identified variant (p. Thy669Cys) was previously described in the heterozygous state within a family affected by WS [24]. Our findings further support the pathogenicity of this variant, as it was identified in both the index case and the younger brother, who also has diabetes. In this family, the consanguineous parents are asymptomatic carriers of the mutation, as the inheritance pattern of WS is autosomal recessive. Based on our findings, patient P7 and the brother, who have been diagnosed with WS, should receive long-term interdisciplinary clinical follow-up involving specialists such as an endocrinologist, diabetologist, ophthalmologist, audiologist and psychologist.

Patient P10 has two heterozygous variants in the *PLIN1* and *TCF7L2* genes. The perilipin 1 (*PLIN1*) gene encodes a protein that regulates lipid metabolism in adipocytes [25]. The *TCF7L2* gene encodes a transcription factor 7 like 2, which plays a key role in blood glucose homeostasis [26]. Genetic mutations in this gene are associated with Familial partial lipodystrophy type 4; an autosomal dominant metabolic disorder characterized by the onset of subcutaneous adipose tissue, insulin-resistant diabetes mellitus, hypertriglyceridemia and hypertension in childhood or young adulthood [27,28]. Other features may include hepatic steatosis and polycystic ovary syndrome (PCOS) [29], as seen in our patient P10 and the sister, who was diagnosed with diabetes at a young age.

Several studies have confirmed the association between PCOS, diabetes and its complications, such as cardiovascular disease [30]. In addition, Jéru et al. have demonstrated that heterozygous variants of the *PLIN1* gene are linked to familial partial lipodystrophy type 4 [31]. However, another study suggested that heterozygous variants in the *PLIN1* gene do not cause familial partial lipodystrophy and should not be classified as disease-causing [32]. This discrepancy may be attributed to recent doubts about the pathogenicity of other *PLIN1* variants found in patients with diabetes, stemming from the absence of lipodystrophy in these individuals and the higher prevalence of *PLIN1* variants in the general population [32]. Our ORVAL analysis showed a potentially pathogenic combination (VarCoPP score = 0.78) between the *PLIN1* and *TCF7L2* genes. Therefore, variants in these two genes contribute to a dual molecular diagnosis: diabetes and PCOS consistent with a diagnosis of Familial partial lipodystrophy type 4 in P10.

### 4.2. Unspecified MFD

Genetic testing for patient P5 revealed the presence of two heterozygous variants in the *MKS1* and *DMPK* genes. The *MKS1* gene encodes a basal body protein required for primary cilia in ciliated epithelial cells. Mutations in this gene are associated with Meckel syndrome type 1 [33]. Most individuals with Meckel syndrome develop liver fibrosis, which is also present in P5. The *DMPK* gene encodes a serine-threonine kinase that is closely related to other kinases involved in the insulin secretion pathway [34], which helps explain the diabetes observed in P5.

The clinical presentation of patient P5 which includes diabetes, hepatic fibrosis and marked asthenia is consistent with that reported in other women aged around 35 years of age, who have mutations in the *DMPK* gene [35]. The ORVAL results did not reveal any significant interaction between the *MKS1* and *DMPK* genes. Therefore, based on these findings, patient P5 is diagnosed with unspecified atypical MFD with Meckel syndrome type 1 comorbidity.

Patient P8 carries two variants in the *ADCY5* (p. Pro10Leu) and *PPP1R3A* (p. Pro756Leu) genes. The *PPP1R3A* gene plays an important role in glycogen synthesis but is not essential for the insulin activation of glycogen synthase. According to the literature, variants in *PPP1R3A* genes are associated with T2D [36] and MFD [6]. The adenylate cyclase 5 (*ADCY5*) gene encodes a member of the family of membrane adenyl cyclase enzymes that mediate insulin secretion signaling pathways. Single nucleotide polymorphisms (SNPs) in this gene may also be linked to T2D [37]. Our results corroborate those of Ivette et al., who identified another variant (rs11708067) in the *ADCY5* gene associated with low neonatal insulin and C-peptide concentrations [38]. Similarly, for the first time in South Asians, researchers have found evidence that SNP alleles in *ADCY5* may increase the risk of T2D [39]. Our ORVAL analysis revealed a potentially true digenic pathogenic combination (VarCoPP score = 0.82, 99% causing of the disease) between the *ADCY5* and *PPP1R3A* genes. In other terms, both genes are essential for the early onset of diabetes in P8.

The same variant found in the *PPP1R3A* gene in P8 was also identified in P9. In addition, genetic analysis revealed another heterozygous variant in the *GIPR* gene, which encodes a glucose-dependent insulinotropic polypeptide receptor. Deficiency in this gene can lead to the development of diabetes. Researchers have identified a set of SNPs in the *GIPR* gene as associated with T2D and gestational diabetes in a large cohort of patients from two ethnic groups [40]. Another previous study highlighted the essential role of *GIPR* in insulin secretion, β-cell proliferation and physiological expansion of β-cell mass [41]. According to the literature, rs10423928 located in the *GIPR* gene, is associated with postprandial blood glucose levels. Zhang et al. report that *GIPR* plays a significant role in regulating food intake and consequently body weight [42]. Considering these results, patient P9 presents unspecified atypical MFD, which require further genetic investigation of MFD in Tunisia for a more accurate diagnosis of diabetes.

We identified two variants, p. Pro152Ala and p. Ser171Thr, located in the *GRB10* and *NEUROG3* genes, respectively, in P11.The *GRB10* (Growth Factor Receptor Bound Protein 10) gene encodes a growth factor receptor-binding protein that interacts with insulin receptors and insulin-like growth factor receptors [43]. Overexpression of certain isoforms of this protein inhibits tyrosine kinase activity leading to growth suppression, as observed in patient P11. A recent study has shown that the rs1800504 polymorphism in the GRB10 gene is associated with blood lipids levels and, subsequently, the risk of coronary heart disease in patients with T2DM [44]. Other studies have linked variants in the *GRB10* gene to variation in plasma glucose levels at birth and in early adulthood [45].

The *NEUROG3* gene encodes a protein Neurogenin 3, a transcription factor involved in neurogenesis. This protein likely acts as a heterodimer with another protein. Related pathways include the regulation of β-cell development and nervous system development. Consequently, the *NEUROG3* gene is associated with hyperglycemia [46].

Our findings align with a recent study that identified *NEUROG3* variants in three unrelated Thai patients with malabsorptive diarrhea, endocrine and renal abnormalities [47]. In addition, a study involving 31 Chinese families with diabetes revealed 32 mutations across six genes including *NEUROG3*, which was a novel causative gene for MODY [48]. Another study confirms that genetic variation in the *NEUROG3* gene may be one of the genetic determinants involved in the pathogenesis of T2D [49]. However, Okada et al. reported no association between *NEUROG3* mutations and T2DM in the Japanese population [50]. Our ORVAL analysis indicated the presence of a potential pathogenic monogenic disease with modifier effect combination (VarCoPP score = 0.80). Collectively, these results support a diagnosis of unspecified atypical MFD in P11.

This study highlights the considerable clinical and genetic heterogeneity of monogenic diabetes in the Tunisian population, driven by genetic, environmental, and behavioral factors. Our results reinforce the emerging concept that oligogenic inheritance plays a role in the phenotypic variability of monogenic diabetes such is the case of patients P3, P8 and P11, emphasizing the need to account for gene–gene interactions in metabolic disease research. So, a deeper understanding of the molecular basis of MFD is crucial for accurate diagnosis and tailored treatment, ranging from dietary changes to targeted therapies, with significant socio-economic implications.

While genetic testing is essential for confirming the diagnosis of monogenic diabetes, the primary barrier to accurate diagnosis occurs earlier in the care pathway—at the stage of clinical suspicion and patient referral. To address the underdiagnosis of monogenic forms of diabetes (MFD), it is crucial to enhance clinical awareness and promote early screening. The key challenge is not the availability of genetic testing but rather identifying which patients should undergo testing. Based on the criteria established in this study, we propose a practical screening approach tailored to the Tunisian context. Simple and accessible clinical indicators can effectively guide physicians in selecting patients most likely to benefit from genetic analysis. Additionally, tools such as the one developed by Welsch et al. (2024) [4] may serve as valuable models to support early detection and improve diagnostic efficiency in routine clinical practice.

The relatively small sample size reflects the rarity and underdiagnosis of monogenic diabetes in Tunisia, partly due to limited access to genetic testing and the strict inclusion criteria requiring detailed biochemical, immunological, and clinical assessments. Nevertheless, each case was extensively characterized, providing meaningful insights into the genetic and clinical diversity of this rare disease.

## 5. Conclusions

Our study emphasizes the considerable clinical and genetic heterogeneity of MFD in Tunisia which limits the effectiveness of targeted gene panels. Therefore, exome sequencing emerges as a more suitable and cost-effective diagnostic approach, especially in middle- and low-income countries. However, future work should include functional validation studies to experimentally confirm the biological impact of the identified variants.

## Figures and Tables

**Table 1:** Anthropometric and clinical characteristics of the 11 suspected MFD patients.

Patient ID	Circumstance of Diabetes Discovery	Age at Diagnosis of Diabetes (Years)	Age at Survey	Treatement	Family Members with Diabetes	Comorbidities (Other Clinical Features)
**P1**	Polyuria, polydipsia FPG = 27.40 mmol/L HbA1c = 9.1%	19	30 s	Insulin (Actrapid 4 U + Insulatard 8 U, 3×/day) than switched to OAD	1	Nephropathy, High levels of T-CHL Gastric problems
**P2**	Polyuria, polydipsia Weight loss FPG = 14.20 mmol/L HbA1c = 14.0%	6	10 s	Insulin (Insulatard 12/4 U; Actrapid 4/2 U)	0	No other clinical signs
**P3**	Polyuria, polydipsia HbA1c = 7.0%	24	30 s	OAD than switched to Insulin (Actrapid 5 U, Insulatard 10 U)	1	Hypoglycemic often at night Acute pyelonephritis, pelviperitonitis, Hypertriglyceridemia anxiety
**P4**	Polyuria, polydipsia FPG = 16.70 mmol/L HbA1c = 10.0%	18	20 s	OAD	2	No other clinical signs
**P5**	Pancreas inflammation	17	20 s	Insulin (Actrapid 14/14/14 U; Insulatard 28/26 U)	0	Inflammation of the pancreas (Stage1) Hepatic thrombosis, Hypothyroidism High TG and T-CHL levels
**P6**	Polyuria, polydipsia FPG = 22.20 mmol/L HbA1c = 13.0%	15	20 s	Insulin than switched to OAD than insulin	0	Allergy and astigmatism
**P7**	Polyuria, polydipsia FPG = 16.70 mmol/L HbA1c = 8.9%	4	20 s	Insulin	2	Partial hearing loss, Bilateral optic atrophy Diabetic neuropathy
**P8**	Polyuria, polydipsia FPG = 21.10 mmol/L HbA1c = 12.0%	23	20 s	OAD	1	No other clinical signs
**P9**	Polyuria, polydipsia FPG = 16.70 mmol/L HbA1c = 9.0%	15	20 s	OAD + insulin	2	Obesity Hypertension
**P10**	Polyuria, polydipsia Nausea FPG = 11.40 mmol/L HbA1c = 10.8%	25	20 s	OAD	3	Decreased visual acuity and migraine PCOS, irregular menstrual cycle with intense pain
**P11**	Polyuria, polydipsia FPG = 13.90 mmol/L	18	20 s	Insulin	2	Delayed staturo-ponderal development, Finger infection, Glaucoma Intellectual disability

FPG, fasting plasma glucose; HbA1c, glycated hemoglobin; TG, triglycerides; T-CHL: Total Cholesterol; OAD, oral anti-diabetes drug; PCOS: Poly-Cystic Ovary Syndrome; U, Units.

**Table 2:** Biochemical and immunological results of the 11 suspected MFD patients.

Patient ID	FPG (mmol/L)	HbA1C (%)	T-CHL (mmol/L)	TG (mmol/L)	HDL (mmol/L)	LDL (mmol/L)	Creatinine (µmol/L)	CRP (mg/L)	C-Peptide Basal (ng/mL)	Pancreatic Antibodies
P1	3.40	9.01	3.35	0.89	1.32	1.55	64.00	0.97	2.20	Two negative
P2	7.80	7.8	4.41	0.50	1.96	2.32	29.00	0.19	0.02	Two negative
P3	13.00	13.8	7.15	1.80	1.29	5.16	49.00	2.32	0.01	Two negative
P4	9.82	6.7	5.1	2.35	1.39	2.64	57.40	4.26	0.01	Two negative
P5	21.06	16.3	5.29	6.77	NA	1.58	59.00	NA	NA	Two negative
P6	17.80	10.0	3.79	0.59	1.39	2.06	50.00	1.78	1.70	Two negative
P7	11.60	7.3	3.24	0.83	1.14	1.72	71.50	4.51	0.01	Three negative
P8	10.70	9.1	3.39	1.33	1.29	1.50	31.20	12.10	1.51	Two negative
P9	5.82	10.4	3.00	1.65	0.72	1.52	37.30	3.10	1.70	Two negative
P10	13.70	9.2	6.13	2.50	1.02	3.97	45.70	NA	1.29	Two negative
P11	13.70	6.5	4.16	0.61	1.24	2.64	38.40	<0.6	0.02	Two negative

T-CHL, total cholesterol; HDL, high-density lipoprotein; LDL, low-density lipoprotein; CRP, C-reactive protein; NA, not available. C-peptide: The connecting peptide is a short 31-amino-acid polypeptide that connects insulin’s A-chain to its B-chain in the proinsulin molecule. Three negative remains negative results for the three pancreatic antibodies.

**Table 4:** Potential pathogenic variant combinations and their effect on disease-causing among suspected MFD patients.

Patient ID	Variants Combination	VarCoPP Score	Prediction of Pathogenicity	Type of Digenic Effect
**P3**	*LMNA*: 4:151791682:T:C *LRBA*: 1:156108510:C:T	0.81	Disease-causing with 99% confidence	True digenic
**P5**	No variant combination	-	-	-
**P8**	*ADCY5*: 3:123167364:G:A *PPP1R3A*: 7:113518880:G:A	0.82	Disease-causing with 99% confidence	True digenic
**P9**	*PPP1R3A*: 7:113518880:G:A *GIPR*: 19:46176123:G:T	0.67	Low pathogenicity	True digenic
**P10**	No variant combination	-	-	-
**P11**	*GRB10*: 7:50737469:G:C *NEUROG3*: 10:71332289:A:T	0.80	Disease-causing with 99% confidence	Monogenic + modifier

**Table 5:** Diabetes subtypes among the 11 Tunisian patients with diabetes.

Patient ID	Typical Clinical Features	Causative Genes	Diabetes Subtype
P1	Nephropathy High levels of CHL Gastric problems	*HNF1A*	MODY_3 *(HNF1A_MODY)*
P2	Absence of other clinical signs	*WFS1*	Isolated diabetes with low penetrance for Wolfram syndrome features
P3	Hypoglycemic often at night Acute pyelonephritis, pelviperitonitis State of anxiety	*LMNA* *LRBA*	Partial familial lipodystrophy type 2
P4	Absence of other clinical signs	*IRS1*	Early onset T2D
P5	Inflammation of the pancreas Liver thrombosis Hypothyroidism High TG and CHL levels	*MKS1* *DMPK*	Unspecified atypical MFD with Meckel Syndrome comorbidity
P6	Astigmatism and allergies	*WFS1*	Isolated diabetes with low penetrance for Wolfram syndrome features
P7	Partial hearing loss Bilateral optic atrophy Diabetic neuropathy	*WFS1*	Wolfram syndrome
P8	Absence of other clinical signs	*PPP1R3A* *ADCY5*	Unspecified atypical MFD
P9	Obesity Hypertension	*PPP1R3A* *GIPR*	Unspecified atypical MFD
P10	Diminished visual acuity and migraine PCOS Irregular menstrual cycle with intense pain	*TCF7L2* *PLIN 1*	Partial familial lipodystrophy type 4
P11	Delayed staturo-ponderal development Finger infection Glaucoma Intellectual disability	*GRB 10* *NEUROG3*	Unspecified atypical MFD

## Data Availability

The original contributions presented in this study are included in the article. Further inquiries can be directed to the corresponding author.
